# Oral and Mucosal Complaints among Institutionalized Care Seniors in Malopolska Voivodeship—The Utility of the Mirror Sliding Test in an Assessment of Dry Mouth

**DOI:** 10.3390/ijerph192113776

**Published:** 2022-10-23

**Authors:** Piotr Michalak, Paulina Polak-Szlósarczyk, Wioletta Dyduch-Dudek, Barbara Kęsek, Elżbieta Zarzecka-Francica, Maria Styrna, Łukasz Czekaj, Joanna Zarzecka

**Affiliations:** 1 Department of Conservative Dentistry with Endodontics Institute of Stomatology, Faculty of Medicine, Medical College, Jagiellonian University, 31008 Krakow, Poland; 2 Department of Periodontology, Clinical Oral Pathology and Prophylaxis, Institute of Stomatology, Faculty of Medicine, Medical College, Jagiellonian University, 31008 Krakow, Poland; 3 Department of Prosthodontics and Orthodontics, Institute of Stomatology, Faculty of Medicine, Medical College, Jagiellonian University, 31008 Krakow, Poland; 4 Municipal Health Centre for Older and Dependent Individuals, 30663 Krakow, Poland

**Keywords:** older people, gerodontology, oral health, dry mouth, long-term care

## Abstract

The purpose of this article was to evaluate reported oral and mucosal complaints among seniors residing in institutionalized 24-h care of the Municipal Center for Older and Dependent People (MHCOD) and the 3-month rehabilitation program of the Daily Medical Care House (DMCH). We evaluated the feasibility of using the dental mirror slidding test to assess dry mouth of seniors. Patients underwent a questionnaire, and clinical examination. The Visual Analogue Scale (VAS) was used to assess pain, Fox’s questionnaire and Challacombe’s scale with Clinical Oral Dryness Score to assess dryness of the mouth, dental mirror slidding test to assess buccal mucosal resistance. Dryness, mucosal burning, impaired taste, food intake are symptoms associated with seniors, and their frequency does not depend on the type of care. The incidence of mucosal burning (Mdn = 4.0, IQR = 4.75, *p* = 0.032) and difficulty in using dental prosthetics (Mdn = 3.0, IQR = 4.00, *p* = 0.010) increase with the length of stay at MHCOD. Seniors are at risk of side effects of polypharmacy, which cause dryness (*p* = 0.036), complaints of lack of saliva (*p* = 0.009) and taste disorders (*p* = 0.041. Seniors with higher levels of dry mouth are more likely to exhibit mucosal burning (*p* = 0.026) and difficulty in taking food (*p* = 0.037). The implementation of the dental mirror slidding test in the scope of the primary care of geriatric examination should be discussed.

## 1. Introduction

The number of dependent elderly people in most parts of the world is expected to increase in the upcoming years [1]. A recently published study in Poland indicates that 39,800 patients were in the care of the Municipal Health Center for Older and Dependent People in 2017, and this represents an increase of 9.04% over 5 years [2]. The oral health of the aging population living in an institutionalized care is a significant challenge, as it is closely related to the general health and quality of life [3].

The population in an institutionalized care consists of those who are particularly vulnerable because they are relatively frail and dependent. Some conditions have consequences for oral health, and a number of medications taken can cause side effects, for example: xerostomia, difficulty swallowing, and taste disorders [4,5]. This results in a reduced quality of life for the aging population [6]. Poor oral health has been suggested to be an additional component of geriatric syndrome and is known as the “new geriatric syndrome.” Therefore, early recognition of oral health problems and referring seniors to a specialist is important [7].

Due to the shortage of personnel and the insufficient training of the carers of elderly in the prevention of oral diseases in Poland most residents of nursing homes are at increased risk for these diseases and their consequences [3,6,8,9]. Lack of or inadequate oral care increases the risk of focal pathologies, for example: pneumonia, cardiovascular disease, diabetes, stroke, osteoporosis, kidney disease, gastrointestinal disease [10,11,12].

The aim of our study was to evaluate the most commonly reported oral and mucosal complaints among seniors residing in the institutionalized care of the Municipal Health Center for Older and Dependent People (MHCOD) and the Daily Medical Care House (DMCH). In addition, we evaluated the utility of the dental mirror sliding test in the examination of dry mouth of senior patients. We hypothesized that the use of this test by nursing staff would make it possible to quickly and inexpensively screen patients and assess the need for patient consultation with a physician. In conducting the analysis, our aim was to explore additional aspects of frailty in the lives of senior patients and to complement our previously published studies [13].

## 2. Materials and Methods

The study was conducted between 11/2017 and 05/2019 and 80 patients have participated. The first group was 50 people in institutionalized care, residents of a Municipal Health Center for Older and Dependent People (MHCOD). Among them were 19 men (38%) and 31 women (62%). The second consisted of 30 participants of a 3-month rehabilitation program of the Daily Medical Care House (DMCH), 9 men (30%), 21 women (70%). There were no statistically significant difference in the number of genders between the groups (*p* = 0.467) Similarly, in terms of age. The mean age of MHCOD men was 66.5 and DMCH 70.6 (*p* = 0.657), while that of women was 78.2 and 72.9 years, respectively (*p* = 0.222) (previously published) [13].

MHCOD is an Independent Public Health Care Facility supervised by the Health Care Department of the City of Krakow. DMCH is a form of activity of this facility, which aims to extend the period of independent living of seniors. In the MHCOD group, patients were cared for 24/7 while participants in the 3-month rehabilitation program lived independently or with their families.

The inclusion criterion for the study was an assessment of cognitive ability by a psychologist using the MMSE scale (Mini-Mental State Examination). The MMSE was evaluated in the following categories: normal > 25 points, suspected cognitive impairment 21–24 points, definite cognitive impairment < 20 points. The study group did not show statistically significant differences in terms of cognitive ability. The MHCOD group had a mean score of 25, while the DMCH group had a mean score of 25.8). Patients had given written consent to participate in the study.

Patients were interviewed and clinically examined by two dentists in a medical office settings, under the light of a headlamp, using a dental mirror and a dental probe [14].

In the interview, patients were asked about oral and mucosal complaints. The respondent could answer YES/NO to each question. The Visual Analogue Scale (VAS) was used to assess pain. To assess dryness of the mouth, Fox’s questionnaire was used [15] and a clinical examination using the Challacombe Scale, with the Clinical Oral Dryness Score (CODS) to assess the severity of dry mouth and treatment needs [16]. In addition, a dental mirror sliding test was performed to assess the resistance of the buccal mucosa while sliding the mirror over its inner surface. The test was performed according to the formula: 1—no resistance, 2—slight resistance, 3—significant resistance. No resistance indicates a normal degree of mucosal lubrication with saliva, significant resistance no or very little saliva covering the mucosa.

The analysis were performed using the statistical language R (version 4.1.1; R Core Team, 2021) on Windows 10 × 64. The relationship of variables on a nominal scale was examined using Fisher’s exact test. The normality of the distributions of the variables on the continuous scale was examined using the Shapiro-Wilk test and was based on the calculated skewness and kurtosis parameters. For independent groups with a normal distribution, the Welch’s *t*-test with Hedges’ g effect size calculation was used. For two independent groups with a non-normal distribution, the Mann-Whitney U test was used with calculation of two-point correlation based on ${\hat{r}}_{biserial}^{rank}$ rank. In the form of a host- hoc test, the Gamesa-Howell test and Dunn’s test were used. Investigation of differences in mean measures of variables with a non-normal distribution with the number of groups of 3 with different variance between groups was carried out based on the Kruskal-Wallis one-way analysis of variance test, χ^2^*_Kruskal-Wallis_*.

The study was conducted with the approval of the Jagiellonian University Bioethics Committee 1072.6120.187.2017.

## 3. Results

The most commonly reported oral complaints were dryness 36%, lack of saliva 30% and pain 13.8% (Table 1), while the mucosal complaints were decrease in saliva 45%, difficulty in taking food 23.8%, and difficulty in using dentures 23.8% (Table 2). Between MHCOD and DMCH patients we found no statistically significant differences in reported complaints. As a result, the groups were analysed collectively for reported complaints.

Patients with complaints of mucosal burning were characterized by a significantly longer stay in MHCOD (Mdn = 4.0; IQR = 4.75) than patients without burning (Mdn = 1.0; IQR = 2.75), *p* = 0.032. Furthermore, patients who had problems with prosthesis use were characterized by a significantly longer stay at MHCOD (Mdn = 3.0; IQR = 4.00) than patients without an identified prosthesis problem (Mdn = 1.0; IQR = 2.00), *p* = 0.010 (Table 3).

The distribution of the types of medications taken did not differ significantly between group of patients. The greatest variation in the frequency of medications taken between groups was observed for antidepressants and for Parkinson’s disease (Table 4).

Welch’s *t*-test showed no significant differences in the number of medications taken between the DMCH group (M = 6.23, SD = 3.13) and the MHCOD group (M = 6.32; SD = 2.34); *p* = 0.896 (Table 5).

As there were no statistically significant differences between the groups, a combined analysis of the relationship between the amount of medications and oral and mucosal complaints was performed. The group of patients who took more medications reported less frequent oral bleeding (M = 6.43 SD = 2.59 vs. M = 2.67 SD = 1.15; *p* = 0.016) while more frequent dryness (M = 7.14 SD = 2.76 vs. M = 5.80; SD = 2.47 *p* = 0.036) and complaints of lack of saliva (M = 7.50 SD = 2.64 vs. M = 5.77 SD = 2.49; *p* = 0.009 (Table 6).

Patients who reported complaints of taste disorder took significantly more medications (M = 9.20; SD = 2.39) than patients without complaints (M = 6.09; SD = 2.56), *p* = 0.041 (Table 7).

Analysis of the results of the Fox’s questionnaire showed no statistically significant differences between the study groups. However, the greatest variation in responses was observed in swallowing problems and the need to chew a gum on a daily basis to eliminate a feeling of dryness in the mouth, which were the least reported by seniors. The complaints reported the most frequently in both groups were the sensation of too small or too much saliva and the need to sip while chewing dry foods (Table 8).

The clinical examination conducted according to the scores included in the Challacombe scale showed a statistically significant correlation concerning only the issue of changes in the gingival structure. In addition to this, variations were observed at the trend level on the issue of lobulated/fissured tongue (Table 9).

The data counted according to the Clinical Oral Dryness Score (CODS) formula did not show differences between the study groups. Among the subjects, as many as 52.5% obtained a score indicating moderate level of oral dryness (DMCH = 50%, MHCOD = 54%) (Table 10).

**Figure 1:**
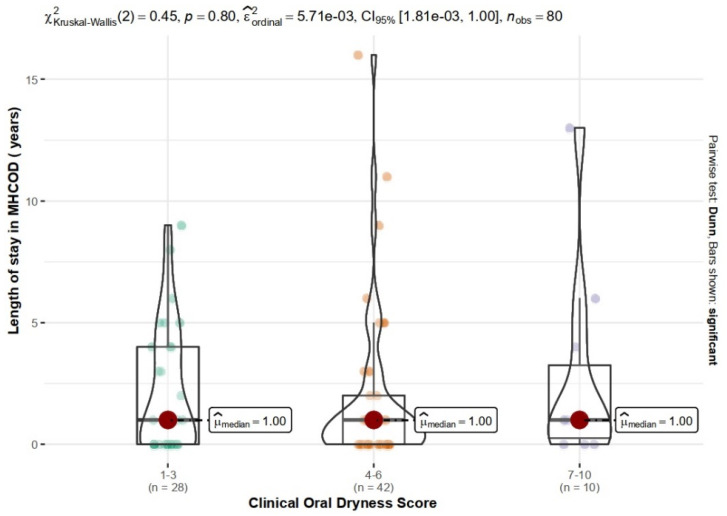
Relationship between the length of stay in MHCOD and CODS.

Analysis of the relationship between the Clinical Oral Dryness Score and the length of the patient’s stay at MHCOD showed no statistically significant relationship (χ^2^_Kruskal-Wallis_(2) = 0.45 *p* = 0.798, ${\hat{e}}_{ordinal}^{2}$ < 0.01) (Figure 1).

An analysis of the relationship between CODS scores and mucosal complaints showed several correlations. There is a significantly higher incidence of mucosal burning in patients with scores of 4–6 compared to those with scores of 1–3, *p* = 0.026. In addition, patients with scores of 7–10 have a significantly higher proportion of food intake problems than patients with scores of 1–3 (50% and 10.7, respectively) (Table 11).

Analysis of the results of sliding mirror test did not show statistically significant differences between the DMCH and MHCOD groups (Table 12). For this reason, it was decided to treat the group collectively in subsequent analyses.

Analysis of the relationship between the results of sliding mirror test and reported complaints revealed several significant relationships.

### 3.1. Decrease in Saliva

Patients with no resistance in the sliding mirror test showed the lowest proportion of subjectively perceived saliva reduction (10.3%). This difference was significant compared to patients with low resistance (51.6%) and compared to patients with high resistance (85%). On top of this, there was a difference between the proportions of subjectively perceived saliva reduction between patients with low and high resistance (Table 13).

### 3.2. Taste Disorders

Patients with no resistance in the sliding mirror test did not show taste disorders (0%). This difference was significant compared to patients with significant resistance (20%) (Table 13).

### 3.3. Difficulty in Taking Food

Patients with no resistance in the sliding mirror test showed the lowest proportion of intake problems (13.8%). This difference was significant compared to patients with high resistance (50%). The same difference was shown between the proportions of patients with low resistance (16.1%) and high resistance (50%) (Table 13).

### 3.4. Oral Pain

**Figure 2:**
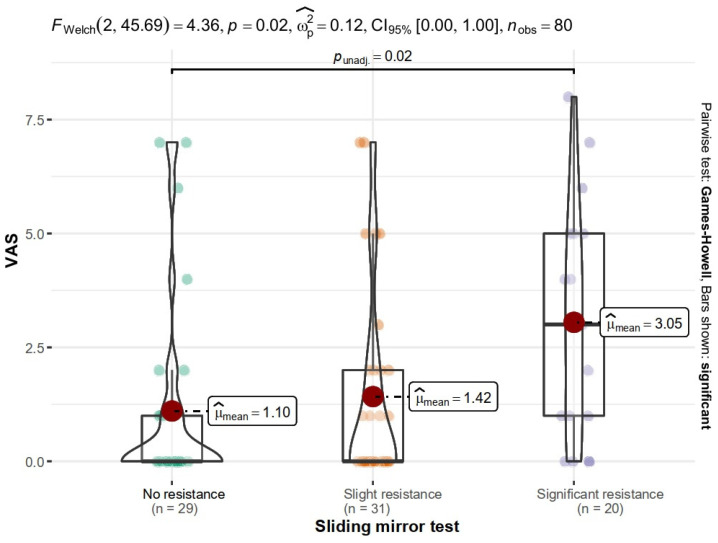
Relationship between VAS and the score of sliding mirror test.

There was a significant relationship of oral pain between the group of patients with no resistance and significant resistance, M = 5.41 and M = 7.70, respectively (*p* = 0.018). Patients with significant resistance had higher VAS scores (Figure 2).

### 3.5. Number of Medications Taken

**Figure 3:**
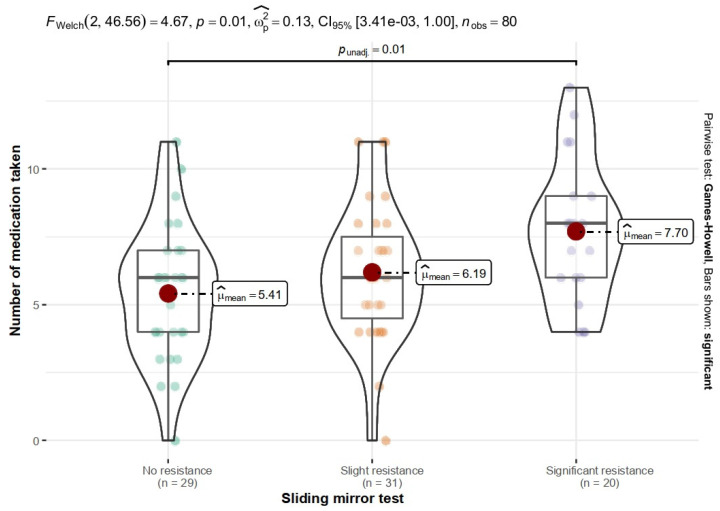
Relationship between the number of medications taken and the score of the sliding mirror test.

There was a significant relationship in the number of medications taken between the group of patients with no resistance in the sliding mirror test (M = 1.10; SD = 2.47) and patients with high resistance (M = 3.05; SD = 2.62), *p* = 0.014. Patients with higher resistance took more medications (Figure 3).

Due to the lack of statistically significant differences in the answers given in the Fox’s questionnaire and the results of the sliding mirror test between the study groups, a comparative analysis of the two studies was performed. This analysis resulted in several significant correlations (Table 14).

It has been noted that:

1. Regardless of the time of day, patients with slight and significant resistance showed more frequent dry mouth.
2. There was a significant difference in the need to hold a glass of water at the bedside between patients with no and slight resistance.
3. Patients with no resistance showed the lowest proportion in a need of drinking water after a dry meal (10.3%). This difference was significant compared to both slight (54.8%) and high resistance (85%) patients.
4. Patients with no resistance were the least likely to have mucosal dryness while eating and thus the least likely to have swallowing disorders.
5. Patients with significant resistance had a need for candy to reduce feelings of dryness.
6. Patients with slight and significant resistance found the amount of saliva too low and show the need to moisten the mouth.

## 4. Discussion

The aim of our study was to evaluate the most commonly reported oral and mucosal complaints among seniors residing in the institutionalized care. We hypothesized that the use of dental mirror test by nursing staff would make it possible to examine seniors and assess the need for consultation with a physician. Our study shows that seniors are at risk for side effects of polypharmacy, which increases the risk of dry mouth and taste disorders. Seniors with higher levels of dry mouth are more likely to exhibit mucosal burning and difficulty taking food. Additionaly these complaints are the most commonly reported. Using the dental mirror sliding test will enable rapid and inexpensive screening of patients for mucosal dryness and prevention of the impact of dry mouth on the quality of life of senior patients.

The aging of the population and the increase in the proportion of people over 65 is a problem that most countries struggle with. There were 90.5 million seniors living in the European Union in 2019. At that time, they represented about 20.3% of the total population. By 2050, it is expected to increase to 129.8 million [17].

The incidence of oral mucosal disease is more common in older people. The incidence of lesions is determined by factors as chronic trauma, oral hygiene and pharmacotherapy [12,18,19]. Oral complaints, dryness, decreased saliva production, impaired taste, swallowing, and problems with the use of prosthetic restorations are significant problems for the older population [20,21,22,23,24,25]. According to the results we presented, these were the symptoms most frequently reported among senior patients. At the same time, it should be mentioned that we did not show differences in reported complaints between patients who underwent different types of care. This is consistent with previously published work [26]. Therefore, it should be understood that the oral health of elderly patients in an institutionalized 24-h care is poor and tends to deteriorate already before they are admitted to this type of facility [27].

### 4.1. Mucosal Burning

The conducted analysis showed that the risk of mucosal burning and difficulties with the use of prosthetic restorations increases with the length of patients stay in the MHCOD. This is probably a consequence of the general health deterioration with age and atrophic changes in the oral mucosa and alveolar bone. This hypothesis may be supported by the results of studies showing that the prevalence of mucosal burning is highest in the 70–79 age group [28]. However, due to the limited 3-month rehabilitation stay of the patients at DHMC, a similar analysis could not be performed in this group. The available bibliography classifies two types of Burning Mouth Syndrome (BMS): primary (true) and secondary. Primary is caused by peripheral or central trigeminal neuropathy. Secondary is a complication of existing local and systemic diseases and medications [29,30]. Oral burning may be associated with diabetes mellitus, anemia, hypothyroidism, gastrointestinal disorders, and Sjogren syndrome. The use of hypotensive drugs that affect the angiotensin-renin system, antiviral drugs, and female hormones may also affect oral burning [24].

### 4.2. Polypharmacy

An analysis of the number of medications taken showed that patients suffer from polypharmacy. Research by the World Human Organization (WHO) indicates that half of older people are at risk of using polypharmacy [31]. The most commonly used definition of this term is taking 5 or more different medications per day [32]. In both study groups, the mean and median values exceeded this value. The relationship between mucosal dryness and taste disorders and the number of medications taken has been the subject of many studies and hypotheses. Patients undergoing polypharmacy are less willing to take care of themselves and use dental services less frequently. This results in poorer oral hygiene [5]. Dry mouth has been reported to depend on the number of drugs, the duration of their use, and the interactions between them, and not specifically on the type of drugs [26,33,34]. We confirmed this hypothesis by performing a sliding mirror test for mucosal dryness. We observed greater mucosal resistance in patients taking a more medications, with no statistically significant correlation with a specific drug group (*p* < 0.05). We further confirmed this by analysing data on the number of drugs and subjective symptoms reported by patients.

### 4.3. Taste Disorders

Multimorbidity and polypharmacy are also risk factors for the development of taste disorders [24]. This is consistent with the results of our study. We found a direct relationship between the number of medications taken and taste disorders. On the other hand, we found no correlation with the place of residence. This contrasts with studies showing that approximately 14 per cent (13.9%) of older people in institutional care and 3.27% of older people living outside report a taste disturbance or loss of taste [35].

The presence of saliva in the mouth is essential for taste perception, which is activated during the initial intake of food [25]. Degeneration of peripheral neurons, central processing abnormalities, and chronic diseases such as diabetes mellitus, Alzheimer’s disease, Parkinson’s disease, kidney failure, chronic type C hepatitis can affect taste perception [36,37,38,39,40,41]. In addition, geriatric patients notice a gradual loss of taste buds, decreased neuromuscular control, and muscle strength resulting in decreased chewing performance [42]. Chronic pain of the oral mucosa has been found to cause taste disorders, as well as taste disorders can cause burning of the oral mucosa [43]. The coexistence of these symptoms was confirmed in our study by using the sliding mirror test. Patients with higher oral mucosal resistance simultaneously showed higher VAS pain scores, as well as more frequent taste disturbances.

### 4.4. Difficulty in Taking Food

Taste disorders have a significant impact on appetite, which can ultimately lead to malnutrition [44]. Our results showed that an increase in the number of medications taken causing the risk of dry mouth, may indirectly influence the risk of eating disorders. Although we did not show differences in medication intake between groups, studies by other authors show that swallowing disorder is one of the most common side effect in the therapy with angiotensin-converting enzyme inhibitors, sedatives, opioids, neuroleptics, anticholinergic drugs in older people [26].

In our study, we showed that the degree of intensity of dry mouth, expressed by the CODS score, is closely related to patients’ complaints of burning mouth and intake disorders. In addition, the performed mirror sliding test showed that as mucosal resistance increases, the frequency of patient-reported intake problems increase. Swallowing dysfunctions are common among older adults, and their prevalence increase with age and frailty. This results in a worsening quality of life for the elderly [45]. Other studies have shown that an increase in mouth pain, expressed on a VAS scale, increases the risk of malnutrition [27]. Furthermore, people with hyposalivation and/or xerostomia have difficulty speaking, chewing, and swallowing, especially dry foods, and they need to sip while eating to improve swallowing [26]. This was confirmed by analysing the responses from the Fox’ questionnaire. We showed that the most frequently reported responses in our study population were saliva disorders that occur simultaneously with the need to sip dry foods.

### 4.5. Dry Mouth

It should be noted that dry mouth is one of the most commonly reported symptoms, occurring in up to 40% of seniors [23,46,47]. This is also demonstrated by the CODS result in our study. This result indicated that dry mouth affects seniors regardless of location and length of stay, with the majority showing medium intensity [16]. The score means that patients should undergo additional diagnostics, require periodic check-ups, and use prophylaxis in the form of sugar-free gum, saliva substitutes, and fluoride. Nevertheless, the Fox’s test results showed that up to 86.7% of DMCH patients and 94% of MHCOD patients do not use chewing gum to control dry mouth. The discrepancies between the information obtained from the patients’ history and clinical examination may be due to the a lack of knowledge among the patients about the prevention options for mucosal dryness and oral diseases. Therefore, further education of nursing staff who can present this information to patients is important. In addition, studies indicate that adequately trained nursing staff is more acceptable by patients [48]. The studies by other authors also indicate that women who have symptoms of bleeding or sore gums, difficulty swallowing liquids or chewing solid foods, and who suffer from multiple chronic diseases are at higher risk of dry mouth [47].

### 4.6. Sliding Mirror Test

By conducting the mirror sliding test, we aimed to see if the information obtained from the test could be used to assess the patient’s clinical condition and the possible need for specialist consultation. The results confirmed that this test can be used to assess dry mouth and related symptoms. This is shown by the correlation between the reduction of saliva reported in the examination and the resistance of the mirror during the test. In addition, we showed correlations between the sliding mirror test result and: taste disorders, problems with food intake, intensity of pain, and number of medications taken. From the analysis of correlations with responses to the Fox’s questionnaire, we obtained correlations that can be used clinically in the care of seniors. Research by other authors indicates the need to develop a questionnaire for assessing oral complaints that can be performed by nursing staff [8,24]. In our opinion, in times of an ageing population a shortage of medical professionals, and rising costs of treatment and care, nursing staff should be trained in the use of the mirror test to screen senior patients for dry mouth and associated symptoms.

It should be noted that our results represent the characteristics of a specific population that lives in the southern part of Poland. Furthermore, due to the limitations of the number of patients in MHCOD, the short period of rehabilitation in DMCH, and the required written consent of seniors who participate in the study, they are based only on a group of 80 people. The homogeneity in terms of MMSE and the lack of a control group that shows significant cognitive impairment may be a limitation of the results presented.

The strengths of our study are the lack of statistically significant differences in age, gender, and cognitive impairment. Additionally, we performed an extensive comparative analysis using the Challacombe, Fox, and mirror test. This allowed us to obtain a lot of clinically relevant information mentioned in the article.

## 5. Conclusions

1. Dryness, taste and food intake impairment and mucosal burning are symptoms associated with seniors, and their frequency does not depend on the type of care. The propability of mucosal burning and difficulty in using dental restorations increases with the length of stay at MHCOD.
2. Senior patients are at risk for side effects of polypharmacy, which increases the risk of dry mouth and taste disorders. Seniors with higher levels of dry mouth are more likely to exhibit mucosal burning and difficulty taking food.
3. The implementation of the sliding mirror test in the scope of the primary care geriatric examination should be discussed. The results confirmed that this test can be used to assess dry mouth and related symptoms. We showed correlations between the sliding mirror test result and: taste disorders, problems with food intake, intensity of pain, and number of medications taken. The use of sliding mirror test will allow the rapid and inexpensive screening of patients for mucosal dryness and prevention of the impact of dry mouth on the quality of life of senior patients.

## Figures and Tables

**Table 1:** The frequency of oral cavity complaints in both groups.

Oral Cavity Complaints	Study Group				*p*	φ_c_
	DMCH		MHCOD			
	No	Yes	No	Yes		
Pain	26 (86.7%)	4 (13.3%)	43 (86%)	7 (14%)	1.000	0.01
Bleeding	28 (93.3%)	2 (6.7%)	49 (98%)	1 (2%)	0.553	0.12
Tooth Mobility	30 (100%)	0	49 (98%)	1 (2%)	1.000	0.09
Halitosis	28 (93.3%)	2 (6.7%)	48 (96%)	2 (4%)	0.628	0.06
Oral burning	28 (93.3%)	2 (6.7%)	50 (100%)	0	0.138	0.21
Excess of saliva	29 (96.7%)	1 (3.3%)	49 (98%)	1 (2%)	1.000	0.04
Dryness	20 (66.7%)	10 (33.3%)	31 (62%)	19 (38%)	0.857	0.05
Swallowing problems	28 (93.3%)	2 (6.7%)	41 (82%)	9 (18%)	0.195	0.16
Lack of saliva	24 (80%)	6 (20%)	32 (64%)	18 (36%)	0.207	0.17
Pain, crackling, skipping in temporomandibular joint (TMJ)	28 (93.3%)	2 (6.7%)	49 (98%)	1 (2%)	0.553	0.12
Other	30 (100%)	0	48 (96%)	2 (4%)	0.525	0.12

Fisher’s exact test.

**Table 2:** The frequency of oral mucosal complaints in both groups.

Oral Mucosal Complaints	Study Group				*p*	φ_c_
	DMCH		MHCOD			
	No	Yes	No	Yes		
General	28 (93.3%)	2 (6.7%)	48 (96%)	2 (4%)	0.628	0.06
Decrease in saliva	17 (56.7%)	13 (43.3%)	27 (54%)	23 (46%)	1.000	0.03
Increase in saliva	29 (96.7%)	1 (3.3%)	48 (96%)	2 (4%)	1.000	0.02
Taste disorder	29 (96.7%)	1 (3.3%)	46 (92%)	4 (8%)	0.645	0.09
Mucosal burning	26 (86.7%)	4 (13.3%)	44 (88%)	6 (12%)	1.000	0.020
Difficulty in taking food	24 (80%)	6 (20%)	37 (74%)	13 (26%)	0.598	0.07
Problem with performing hygiene procedures	28 (93.3%)	2 (6.7%)	43 (86%)	7 (14%)	0.471	0.11
Difficulty in using dentures	26 (86.7%)	4 (13.3%)	35 (70%)	15 (30%)	0.109	0.19

Fisher’s exact test.

**Table 3:** Relationship between length of stay at MHCOD and reported oral mucosal complaints.

Oral Mucosal Complaints	Lack of Complaints		Presence of Complaints		W_Mann-Whitney_	*p*	95% CI	${\hat{r}}_{biserial}^{rank}$
	n	Mdn (IQR)	n	Mdn (IQR)				
General	76	1.0 (3.25)	4	0.5 (1.5)	179.0	0.540	−0.38–0.64	0.18
Decrease in saliva	44	1.0 (2.25)	36	1.0 (4.0)	689.5	0.300	−0.37–0.13	−0.13
Increase in saliva	77	1.0 (3.0)	3	5.0 (3.0)	79.00	0.340	−0.76–0.33	−0.32
Taste disorder	75	1.0 (3.0)	5	1.0 (4.0)	150.5	0.450	−0.62–0.31	−0.20
Mucosal burning	70	1.0 (2.75)	10	4.0 (4.75)	208.0	0.032	−0.67–−0.05	−0.41
Difficulty in taking food	61	1.0 (3.0)	19	1.0 (5.0)	465.0	0.180	−0.46–0.10	−0.20
Problem with performing hygiene procedures	71	1.0 (3.0)	9	1.0 (4.0)	251.0	0.280	−0.55–0.18	−0.21
Difficulty in using dentures	61	1.0 (3.0)	19	2.0 (4.0)	360.5	0.010	−0.60–−0.10	−0.38

Mann-Whitney test.

**Table 4:** Relationship between the type of medication taken and the place of residence.

Type of Medications	Study Group				*p*	φ_c_
	DMCH		MHCOD			
	No	Yes	No	Yes		
Anticholinergics	29 (96.7%)	1 (3.3%)	46 (92%)	4 (8%)	0.645	0.09
Antihistamines	26 (86.7%)	4 (13.3%)	47 (94%)	3 (6%)	0.416	0.13
Antihypertensive	9 (30%)	21 (70%)	14 (28%)	36 (72%)	1.000	0.02
For Parkinson’s disease	29 (96.7%)	1 (3.3%)	41 (82%)	9 (18%)	0.081	0.22
Cytotoxic	29 (96.7%)	1 (3.3%)	47 (94%)	3 (6%)	1.000	0.06
Sedative	12 (40%)	18 (60%)	25 (50%)	25% (50%)	0.524	0.10
Relaxants	28 (93.3%)	2 (6.7%)	44 (88%)	6 (12%)	0.703	0.086
Antidepressants	23 (76.7%)	7 (23.3%)	27 (54%)	23 (46%)	0.074	0.23
Anticoagulants	21 (70%)	9 (30%)	37 (74%)	13 (26%)	0.797	0.04
Bone resorption inhibitors	29 (96.7%)	1 (3.3%)	48 (96%)	2 (4%)	1.000	0.02
Other	6 (20%)	24 (80%)	12 (24%)	38 (76%)	0.786	0.05

Fisher’s exact test.

**Table 5:** Number of medications taken by patients in both groups.

	Study Group	n	M	SD	Mdn	IQR	Min	Max	Sk.	Kurt.	W	*p*
**Number of medications**	DMCH	30.00	6.23	3.13	6.00	3.75	0.00	13.00	0.24	−0.57	0.97	0.07
	MHCOD	50.00	6.32	2.34	6.00	3.75	0.00	12.00	0.14	0.05		

Fisher’s exact test.

**Table 6:** Relationship between oral cavity complaints and the number of medications.

Oral Cavity Complaints	No Complaints		Presence of Complaints		t_Welch_	*p*	95% CI	${\hat{g}}_{\mathrm{Hedges}}$
	n	M (SD)	n	M (SD)				
Pain	69	6.2 (2.35)	11	7.0 (4.12)	−0.65	0.530	−0.92–0.48	−0.23
Bleeding	77	6.43 (2.59)	3	2.67 (1.15)	5.16	0.016	0.18–2.47	1.33
Tooth mobility	79	6.25 (2.64)	1	9.00 (0)	-	-	-	-
Halitosis	76	6.2 (2.57)	4	8.0 (3.92)	−0.91	0.430	−1.29–0.54	−0.40
Burning	78	6.29 (2.66)	2	6.0 (2.86)	0.15	0.910	−0.07–0.08	0.01
Excess of saliva	78	6.19 (2.56)	2	10.0 (4.24)	−1.26	0.420	−0.07–0.03	−0.03
Dryness	51	5.80 (2.47)	29	7.14 (2.76)	−2.16	0.036	−0.97–−0.03	−0.50
Swallowing problems	69	6.36 (2.57)	11	5.82 (3.19)	0.54	0.600	−0.47–0.82	0.18
Lack of saliva	56	5.77 (2.49)	24	7.50 (2.64)	−2.74	0.009	−1.15–−0.16	−0.66
Pain, crackling, skipping in TMJ	77	6.14 (2.57)	3	10.0 (1.73)	−3.70	0.051	−2.22–0.01	−1.12
Other	78	6.27 (2.67)	2	7.00 (1.41)	−0.70	0.600	−0.26–0.14	−0.07

Welch’s *t*-test.

**Table 7:** Relationship between oral mucosal complaints and the number of medications.

Oral Mucosal Complaints	No Complaints		Presence of Complaints		t_Welch_	*p*	95% CI	${\hat{g}}_{\mathrm{Hedges}}$
	n	M (SD)	n	M (SD)				
General	76	6.13 (2.59)	4	9.25 (2.06)	−2.91	0.050	−1.99–0.00	−1.02
Decrease in saliva	44	5.61 (2.32)	36	7.11 (2.81)	−2.56	0.013	−1.02–−0.12	−0.57
Increase in saliva	77	6.23 (2.67)	3	7.67 (1.53)	−1.54	0.240	−1.07–0.25	−0.44
Taste disorder	75	6.09 (2.56)	5	9.20 (2.39)	−2.80	0.041	−1.98–0.04	−1.04
Mucosal burning	70	6.06 (2.51)	10	7.90 (3.14)	−1.78	0.100	−1.30–0.12	−0.60
Difficulty in taking food	61	6.10 (2.46)	19	6.89 (3.16)	−1.01	0.320	−0.81–0.27	−0.27
Problem with performing hygiene procedures	71	6.25 (2.63)	9	6.56 (2.88)	−0.30	0.770	−0.76–0.56	−0.10
Difficulty in using dentures	61	6.08 (2.72)	19	6.95 (2.34)	−1.35	0.19	−0.82–0.16	−0.33

Welch’s *t*-test.

**Table 8:** The results of the Fox’s questionnaire in both groups.

Fox’s Questionnaire	Study Group				*p*	φ_c_
	DMCH		MHCOD			
	No	Yes	No	Yes		
1. Do you experience mouth dryness during the night or upon waking up?	17 (56.7%)	13 (43.3%)	29 (58%)	21 (42%)	1.000	0.01
2. Do you experience mouth dryness during the day?	19 (63.3%)	11 (36.7%)	28 (56%)	22 (44%)	0.681	0.07
3. Do you keep a glass of water next to your bed?	19 (63.3%)	11 (36.7%)	31 (62%)	19 (38%)	1.000	0.01
4. Do you drink fluids while swallowing dry foods?	17 (56.7%)	13 (43.3%)	26 (52%)	24 (48%)	0.862	0.05
5. Do you experience mouth dryness during meals?	18 (60%)	12 (40%)	32 (64%)	18 (36%)	0.905	0.04
6. Do you experience problems with swallowing foods?	22 (73.3%)	8 (26.7%)	41 (82%)	9 (18%)	0.405	0.10
7. Do you use chewing gum on a daily basis to eliminate a feeling of mouth dryness?	26 (86.7%)	4 (13.3%)	47 (94%)	3 (6%)	0.416	0.13
8. Do you use hard fruit or mint candies on a daily basis to eliminate a feeling of mouth dryness?	22 (73.3%)	8 (26.7%)	40 (80%)	10 (20%)	0.583	0.08
9. Do you perceive the volume of saliva in your mouth as too small/excessive, or do you just not notice it?	14 (46.7%)	16 (53.3%)	24 (48%)	26 (52%)	0.817	0.05
10. Do you need to moisten your mouth frequently?	18 (60%)	12 (40%)	33 (66%)	17 (34%)	0.764	0.06

Fisher’s exact test.

**Table 9:** The results of Challacombe Test in both groups.

Challacombe Test	Study Group				*p*	φ_c_
	DMHC		MHCOD			
	No	Yes	No	Yes		
1. Mirror sticks to buccal mucosa	15 (50%)	15 (50%)	24 (48%)	26 (52%)	1.000	0.02
2. Mirror sticks to tongue	15 (50%)	15 (50%)	16 (32%)	34 (68%)	0.173	0.18
3. Saliva frothy	23 (76.7%)	7 (23.3%)	40 (80%)	10 (20%)	0.781	0.04
4. No saliva pooling in floor of mouth	11 (36.7%)	19 (63.3%)	20 (40%)	30 (60%)	0.953	0.03
5. Tongue shows generalised shortened papillae (mild depapillation)	8 (26.7%)	22 (73.3%)	19 (38%)	31 (62%)	0.427	0.12
6. Altered gingival structure (i.e., smooth)	20 (66.7%)	10 (33.3%)	17 (34%)	33 (66%)	0.009	0.32
7. Glassy appearance of oral mucosa, especially palate	20 (66.7%)	10 (33,3%)	33 (66%)	17 (34%)	1.000	0.01
8. Tongue lobulated/fissured	14 (46.7%)	16 (53.3%)	13 (26%)	37 (74%)	0.099	0.21
9. Cervical caries (more than 2 teeth)	28 (93.3%)	2 (6.7%)	41 (82%)	9 (18%)	0.195	0.16
10. Debris on palate or sticking to teeth	18 (60%)	12 (40%)	38 (76%)	12 (24%)	0.141	0.17

Fisher’s exact test.

**Table 10:** The results of Clinical Oral Dryness Score in both groups.

Study Group	Clinical Oral Dryness Score				df	*p*	V
	1–3	4–6	7–10	In Total			
DMCH	12 (40%)	15 (50%)	3 (10%)	30	2	0.755	0.09
MHCOD	16 (32%)	27 (54%)	7(14%)	50			
In total	28 (35%)	42 (52.5%)	10 (12.5%)	80			

Fisher’s exact test.

**Table 11:** Relationship between CODS and oral mucosal complaints.

Oral Mucosal Complaints	No Complaints			Presence of Complaints			χ^2^	V	*p*
	n_1–3_	n_4–6_	n_7–10_	n_1–3_	n_4–6_	n_7–10_			
General	28	39	9	0	3	1	2.41	0.17	0.240
Decrease in saliva	18	23	3	10	19	7	3.51	0.21	0.171
Increase in saliva	26	41	10	2	1	0	1.50	0.14	0.716
Taste disorders	28	38	9	0	4	1	2.88	0.19	0.253
Mucosal burning	28	34	8	0	8	2	6.16	0.28	0.026
Difficulty in taking food	25	31	5	3	11	5	6.57	0.29	0.037
Problem with performing hygiene procedures	27	37	7	1	5	3	5.19	0.26	0.059
Difficulty in using dentures	20	33	8	8	9	2	0.56	0.08	0.757

Fisher’s exact test and post-hoc test.

**Table 12:** The results of sliding mirror test in both groups.

Group	Sliding Mirror Test				df	*p*	V
	No Resistance	Slight Resistance	Significant Resistance	In Total			
DMCH	13 (43.3%)	11 (36.7%)	6 (20%)	30	2	0.549	0.12
MHCOD	16 (32%)	20 (40%)	14 (28%)	50			
In total	29 (36.2%)	31 (38.8%)	20 (25%)	80			

Fisher’s exact test.

**Table 13:** Analysis of the relationship between mucosal complaints and the score of the mirror test in the study groups.

Oral Mucosal Complaints	No Complaints			Presence of Complaints			χ^2^	V	*p*
	n_no resistance_	n_slight resistnace_	n_significant resistance_	n_no resistance_	n_slight resistnace_	n_significant resistance_			
General	29	30	17	0	1	3	5.94	0.27	0.081
Decrease in saliva	26	15	3	3	16	17	27.55	0.59	<0.001
Increase in saliva	28	31	18	1	0	2	3.38	0.21	0.175
Taste disorders	29	30	16	0	1	4	8.87	0.33	0.019
Mucosal Burning	28	17	15	1	4	5	5.03	0.25	0.072
Difficulty in taking food	25	26	10	4	5	10	10.19	0.36	0.006
Problem with performing hygiene procedures	27	28	16	2	3	4	2.16	0.16	0.354
Difficulty in using dentures	25	22	14	4	9	6	2.50	0.18	0.287

Fisher’s exact test and post-hoc test.

**Table 14:** Analysis of the relationship between the results of the Fox’s test and the sliding mirror test.

Complaints	No Complaints			Presence of Complaints			χ^2^	V	*p*
	n_no resistance_	n_slight resistance_	n_signifacant resistance_	n_no resistance_	n_slight resistance_	n_significant resistance_			
Fox1	26	15	5	3	16	15	21.99	0.52	<0.001
Fox2	26	17	4	3	14	16	24.02	0.55	<0.001
Fox3	26	20	4	3	11	16	24.59	0.55	<0.001
Fox4	26	14	3	3	17	17	28.04	0.59	<0.001
Fox5	28	14	8	1	17	12	22.64	0.53	<0.001
Fox6	27	25	11	2	6	9	10.38	0.36	0.010
Fox7	28	29	16	1	2	4	4.40	0.23	0.144
Fox8	28	23	11	1	8	9	12.04	0.39	0.001
Fox9	25	11	4	4	20	16	25.02	0.56	<0.001
Fox10	27	19	5	2	12	15	23.89	0.55	<0.001

Fisher’s exact test and post-hoc test.

## Data Availability

The data that support the findings of this study are available from the corresponding author (P.M.), upon reasonable request.
